# Oxygen Vacancy Mediated Band-Gap Engineering via B-Doping for Enhancing Z-Scheme A-TiO_2_/R-TiO_2_ Heterojunction Photocatalytic Performance

**DOI:** 10.3390/nano13050794

**Published:** 2023-02-21

**Authors:** Changqing Liu, Chenggang Xu, Wanting Wang, Long Chen, Xu Li, Yuanting Wu

**Affiliations:** School of Material Science and Engineering, Shaanxi Key Laboratory of Green Preparation and Functionalization for Inorganic Materials, Shaanxi University of Science and Technology, Xi’an 710021, China

**Keywords:** Z-scheme heterojunction, anatase TiO_2_/rutile TiO_2_, band structure, oxygen vacancy

## Abstract

Fabrication of Z-scheme heterojunction photocatalysts is an ideal strategy for solving environmental problems by providing inexhaustible solar energy. A direct Z-scheme anatase TiO_2_/rutile TiO_2_ heterojunction photocatalyst was prepared using a facile B-doping strategy. The band structure and oxygen-vacancy content can be successfully tailored by controlling the amount of B-dopant. The photocatalytic performance was enhanced via the Z-scheme transfer path formed between the B doped anatase-TiO_2_ and rutile-TiO_2_, optimized band structure with markedly positively shifted band potentials, and the synergistically-mediated oxygen vacancy contents. Moreover, the optimization study indicated that 10% B-doping with the R-TiO_2_ to A-TiO_2_ weight ratio of 0.04 could achieve the highest photocatalytic performance. This work may provide an effective approach to synthesize nonmetal-doped semiconductor photocatalysts with tunable-energy structures and promote the efficiency of charge separation.

## 1. Introduction

The technology of semiconductor advanced oxidation processes (AOPs) has shown great potential in the degradation of organic pollutants in water since it was developed in 1976 [1]. As a typical n-type semiconductor material, titanium dioxide (TiO_2_), has been widely used in the field of photocatalytic degradation due to its unique physical and chemical properties [2,3,4]. However, TiO_2_ only responds to the ultraviolet part (4%) of sunlight due to its wide band gap (3.2 eV), and shows low carrier-separation efficiency (≤10%) and weak redox capability, severely restricting its further application [5,6,7].

Among various strategies to solve these issues, the design and fabrication of heterojunctions are promising due to their merits in separating charge carriers and in coupling the advantages of each component [8,9]. Because they are always constructed with two components using a staggered band structure, the common Type-Ⅱ heterojunctions are usually formed. In this mode, the photogenerated electrons in the component having a higher CB position migrate to the component with a lower CB position, while the holes migrate in the reverse direction. Thus, the recombination of charge carriers is suppressed, and the spatial separation of charge carriers is promoted [10,11,12]. Compared with the traditional type Ⅱ heterojunction, the typical direct Z-scheme heterojunction has the same band-structure configuration but a distinctly different charge-carrier transfer mode. In this mode, the electrons in the CB of the component with the higher CB position (stronger reduction abilities) and the holes in the VB of the component with the lower VB position (stronger oxidation abilities) are preserved, while the electrons and holes with inferior redox powder recombine [13,14]. Therefore, the Z-scheme heterojunction can not only effectively separate charge carriers, but also retain the highest redox potential of each individual component, which helps to obtain more active free radicals (•O_2_^−^ and •OH) to participate in subsequent surface redox reactions [15,16].

To date, various TiO_2_ based Z-scheme heterojunctions have been reported to have improved photoelectrochemical properties, including NiO/TiO_2_ [17], MoS_2_/TiO_2_ [18], Co_3_O_4_/TiO_2_ [19], WO_3_/TiO_2_ [20], Cu_2_O/TiO_2_ [21], etc. However, preparation of the direct Z-scheme heterojunction remains a great challenge, for which the identification of suitable semiconductors and the construction of high-quality heterointerfaces still need to be addressed [22]. For most of the work that simply combines two or more materials, the interfacial resistance between the two phases will restrict charge transfer and affect their stability [23]. Recently, the fabrication of efficient phase junctions has been shown to be an effective way to promote charge separation, and to lead to enhanced photocatalytic activity [24,25,26]. Furthermore, for a TiO_2_ based phase junction, anatase TiO_2_ (A-TiO_2_) and rutile TiO_2_ (R-TiO_2_) can be generated in-situ during the formation of TiO_2_ in a sol-gel process by B-doping [27], implying the potential to form an A-TiO_2_/R-TiO_2_ contacted intact heterointerface.

In addition, recombination at the heterojunction that resulted from the mismatched band alignment is highly detrimental to the efficiency of the photocatalytic process. Also, to form a high-efficiency Z-scheme photocatalyst, in addition to the staggered band structure of the two contacted semiconductors, the oxidation component should possess a lower VB position to display strong oxidation ability, while the other reduction component should have a higher CB position to exhibit strong reduction ability [13,28]. Therefore, engineering the interface and adjusting the band-alignment of the two components are critical for enhanced photocatalytic performance. Furthermore, the introduction of oxygen vacancies (Vo) has been reported to work as an efficient polarization strategy to improve the unsatisfied charge carriers separation during the photocatalytic process, which is critical to restrict the photocatalytic performance of semiconductors [29,30,31]. However, because they can also work as recombination centers, it is a double-edged sword to introduce Vo into the photocatalyst for charge carriers separation [32]. Thus, reasonably designed and constructed Vo concentrations are essential to enhance photocatalytic performance.

Today, it is well known that doping can produce oxygen vacancies in nanostructured TiO_2_, and the content of oxygen-vacancy defects can also be adjusted by the amount of B-doping [33,34,35]. However, the challenge lies in the choice of the synthesis route to control or tune the formation of oxygen-vacancy defects. In addition, boron doping has also been proved to be a feasible approach to modify the phase content of TiO_2_ to form an A-TiO_2_/R-TiO_2_ heterojunction [36]. Niu et al. demonstrated that the amount of B-doping could be optimized to prepare B-doped TiO_2_ as an efficient visible-light-driven photocatalyst [27]. Wang et al. synthesized the B-doped TiO_2_ with a tunable anatase/rutile ratio and proposed that the formed type-Ⅱ phase junction facilitated the separation of charge carriers [37]. However, there are no studies on Z-scheme R–TiO_2_/A–TiO_2_ photocatalysts that operate via synergistic regulation of the anatase/rutile ratio and the formation of oxygen vacancy defects. Moreover, the underlying regulation mechanism, including the essential relation between Vo tuning, the A-TiO_2_/R-TiO_2_ junction, and photocatalytic activity, still needs to be explored [38].

Hence, in this study, we developed an effective approach to in situ formation of a Z-scheme B-doped A-TiO_2_/R-TiO_2_ phase junction with adjustable band structures and oxygen vacancies. The photodegradation activity was enhanced by the Z-scheme charge transfer pathway formed among the phase junction, the markedly positively shifted band potentials, and the synergistically optimized oxygen vacancy defect contents. Moreover, the formation mechanism of the Z-scheme transfer path and the regulating mechanisms of the band structure and oxygen vacancy are studied in detail.

## 2. Materials and Methods

### 2.1. Chemicals

Commercially available titanium sulfate (Ti(SO_4_)_2_, CP) and boric acid (H_3_BO_3_, AR) were purchased from Sinopharm Chemical Reagent Co., Ltd., Shanghai, China. Glucose (C_6_H_12_O_6_, AR), hydrogen peroxide (H_2_O_2_, 35 wt%), acetylacetone (Hacac, AR), and ethanol were obtained from National Reagent Company, Beijing, China. All reagents were used as received.

### 2.2. Preparation of the Catalysts

All samples were prepared by calcinating the prepared precursors. The precursor was obtained by fully mixing Ti, boron, and carbon-precursor solution, dried at 80 °C for 48 h, and then the precursor was heat-treated at 800 °C for 2 h at 5 °C/min in a muffle oven. In our work, the low-content carbon precursor was applied to form an oxygen-deficient environment by reacting with oxygen in the air during the heat-treatment, to incorporate oxygen vacancies into the prepared titanium oxide-based catalysts.

To be specific, the Ti precursor solution was obtained by dropwise addition of H_2_O_2_ (2 mL) and Hacac (5 mL) to Ti(SO_4_)_2_ (0.025 M) ethanol solution (40 mL). The boron precursor solution was obtained by dispersing H_3_BO_3_ in 20 mL of ethanol under magnetic stirring. C_6_H_12_O_6_ (0.50 g) was dissolved in deionized water (20 mL) to prepare the carbon precursor solution. Specifically, the content of H_3_BO_3_ was established based on the molar ratio of boron to Ti atoms, which was x% B-TiO_2_, i.e., 0%, 4%, 8%, 10%, 12% and 14%, respectively. The obtained samples were marked as pure-TiO_2_, 4% B-TiO_2_, 8% B-TiO_2_, 10% B-TiO_2_, 12% B-TiO_2_ and 14% B-TiO_2_, respectively.

### 2.3. Characterizations

The crystal structure was analyzed by X-ray diffraction (XRD, D/max-2200PC, Rigaku, Tokyo, Japan) with Cu Kα radiation. Morphology and microstructures were characterized by scanning electron microscopy (SEM, Verios 460, FEI, Hillsboro, OR, USA), transmission electron microscope (TEM, Tecnai G2 F20 S-TWIN, FEI, Hillsboro, OR, USA), high resolution TEM (HRTEM), and selected-area electron diffraction (SAED). The element composition was identified by X-ray photoelectron spectroscopy (XPS, AXIS SUPRA, Kratos, UK). The absorbance of the catalyst was analyzed by applying a UV-Vis-NIR spectrophotometer (UV-Vis-NIR DRS, Cary 5000, AGILENT, Santa Clara, CA, USA). The fluorescence spectrophotometer detected the photoluminescence (PL, F-4600, Rigaku, Japan) excited at 300 nm. The absorption spectra were measured on a UV-Vis spectrophotometer (UV2800-A, UNICO, Shanghai, China). Electron paramagnetic resonance (EPR) measurements were performed on a Bruker A300 system, during which DMPO (5,5-dimethyl-1-pyrroline N-oxide) was used to capture the signal. The photocurrent (TP), Mott–Schottky (M-S), and electrochemical impedance spectrum (EIS) measurements were evaluated using a electrochemical workstation (CHI760D, CorrTest, Wuhan, China) in a standard three-electrode system. The working electrode was prepared by applying an as-prepared catalyst on the FTO glass substrate.

### 2.4. Evaluation of Photodegradation Activity

The RhB photodegradation activity of the catalysts under simulated sunlight irradiation is described as follows. Firstly, 30 mL of RhB solution (10 mg/L) was made, followed by the addition of 30 mg of the catalyst. After stirring for 15 min in the dark, the prepared solution was illuminated for 90 min using a 300 W Xe lamp with a wavelength in the range of 190–1100 nm as the light source. At regular intervals of 30 min, 5 mL of the solution was collected and tested by a UV-Vis spectrophotometer (UV2800-A, UNICO, Shanghai, China) with wavelength in the range of 200–800 nm, to analyze the residue RhB concentration according to the corresponding peak for the RhB centered at 554 nm under illumination.

## 3. Results and Discussion

### 3.1. Microstructure of the Catalysts

**Figure 1:**
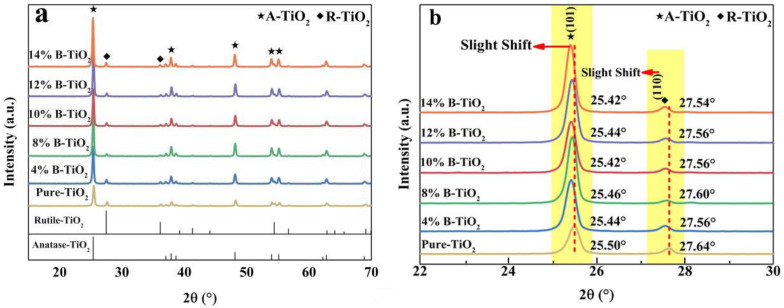
(**a**) XRD patterns of the prepared catalysts, and (**b**) locally-magnified diagrams of the (101) peak of A-TiO_2_ and the (110) peak of R-TiO_2_.

Figure 1a shows the phase composition of the prepared samples. For all samples, only peaks ascribed to rutile TiO_2_ (R-TiO_2_) (PDF 76-1938) and anatase TiO_2_ (A-TiO_2_) (PDF 83-2243) can be detected without the appearance of other phases [39,40]. Furthermore, from the magnified XRD patterns (Figure 1b), the (101) diffraction peak of A-TiO_2_ and (110) diffraction peak of R-TiO_2_ show an obvious shift to the lower 2θ value with increasing introduced boron content. Because the B^3+^ cations (27 pm) are smaller than the Ti^4+^ cations (60.5 pm), the resulting lattice shrinkage would cause the gradual shift of the diffraction peaks [41]. Therefore, these results indicate the successful introduction of boron into TiO_2_ and the substitution of B^3+^ in the crystal lattices of TiO_2_. In addition, the weight ratios of the R-TiO_2_ to A-TiO_2_ are calculated to be 0.1, 0.09, 0.05, 0.04, 0.06, and 0.07, respectively.

**Figure 2:**
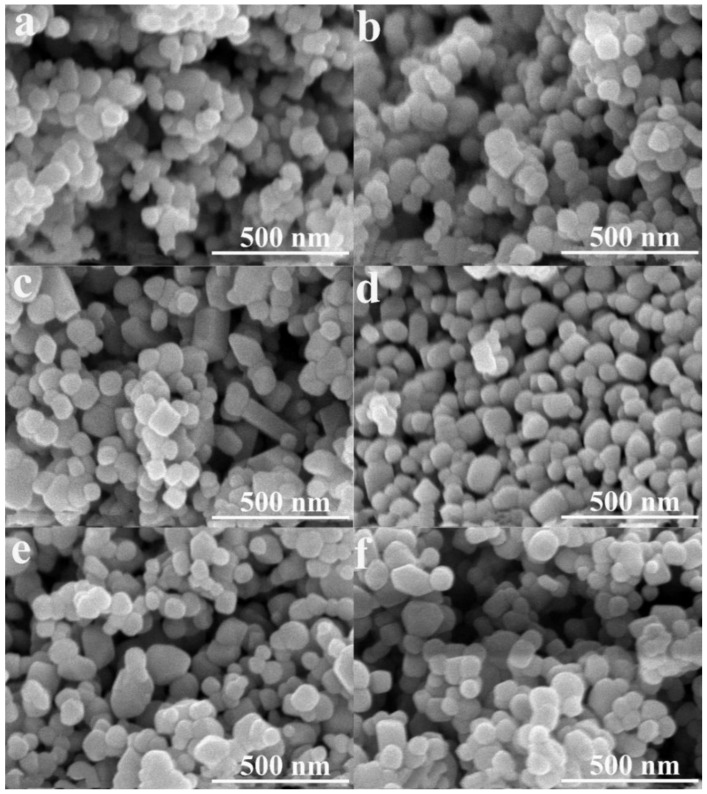
SEM images of all the prepared catalysts: (**a**) pure-TiO_2_, (**b**) 4% B-TiO_2_, (**c**) 8% B-TiO_2_, (**d**) 10% B-TiO_2_, (**e**) 12% B-TiO_2_, (**f**) 14% B-TiO_2_.

**Figure 3:**
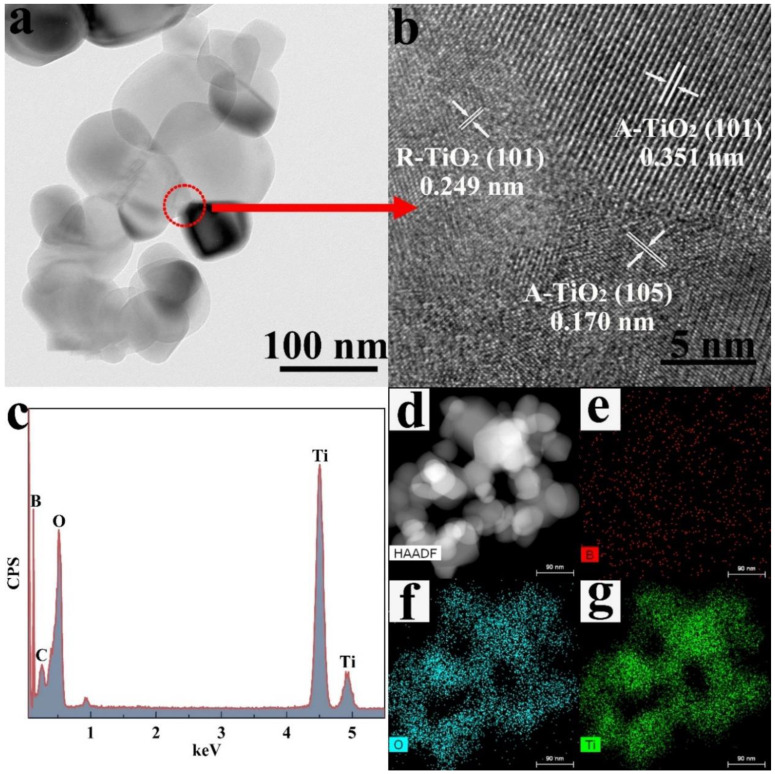
(**a**) Low-resolution TEM images, (**b**) high-resolution lattice images, (**c**) EDS, (**d**) HAADF and EDS elemental mapping images of (**e**) B, (**f**) O, (**g**) Ti for the sample 10% B-TiO_2_.

Figure 2 shows the morphologies of the as-prepared catalysts, and the corresponding particle size statistics are shown in Figure S1. All samples show a uniform and fine particle distribution with an average particle size of about 75 nm. For the pure-TiO_2_ sample (Figure 2a), the particles are in spherical morphologies. Whereas, for the 14% B-TiO_2_ sample (Figure 2f), particles in spherical, ellipsoidal, and rhombic morphologies appeared. Moreover, microstructures of the 10% B-TiO_2_ sample investigated using TEM and HRTEM were shown in Figure 3. The 10% B-TiO_2_ sample shows a similar morphology to that of the 14% B-TiO_2_ sample, including spherical, ellipsoidal and rhombic morphologies. In the HRTEM images (Figure 3b), lattice fringes with the spacing of 0.249, 0.351, and 0.170 nm correspond to the (101) plane of R-TiO_2_, the (101) and the (105) plane of A-TiO_2_ [42,43], respectively, from which the fine-contacted interface between these two phases can be observed. Furthermore, the EDS result in Figure 3c–g reveal the uniform distribution of elements B, Ti, and O, confirming the successful introduction of element B and the formation of the A-TiO_2_/R-TiO_2_ heterostructure.

**Figure 4:**
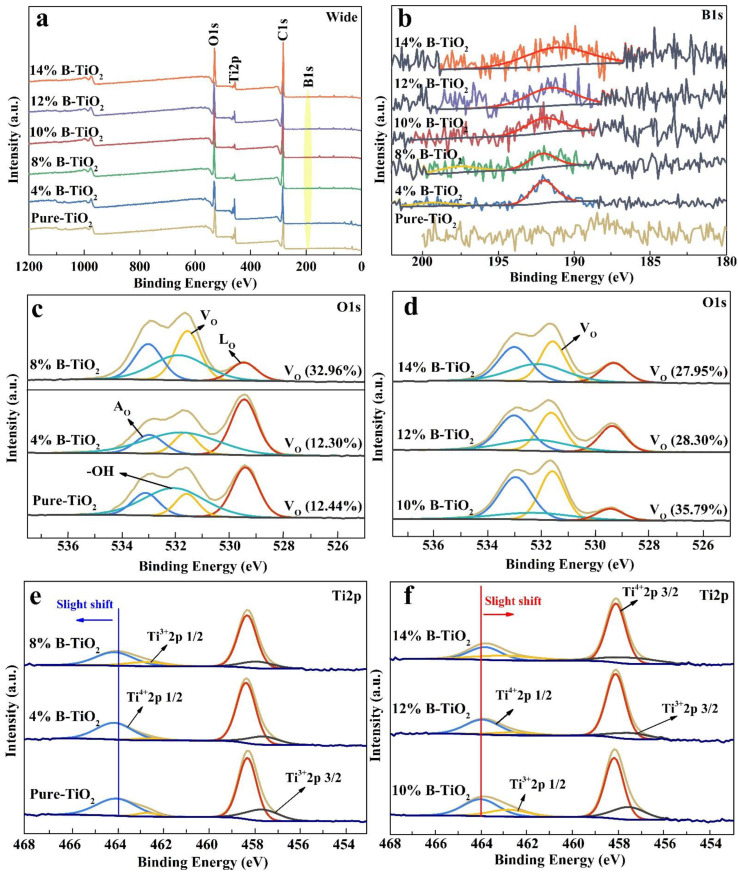
(**a**) XPS survey spectra, (**b**) high-resolution B_1s_ XPS spectra, (**c**,**d**) high-resolution O_1s_ XPS spectra, (**e**,**f**) high-resolution Ti_2p_ XPS spectra of all the prepared catalysts.

XPS analysis was applied to find out the concentrations of defect states (oxygen vacancy) of TiO_2_ after the introduction of B (Figure 4). In the survey spectra (Figure 4a), besides the C calibration element, B, O, and Ti elements are detected for all the samples. Figure 4b shows the XPS spectra of B_1s_, in which a broad peak, centered at around 191 eV ascribed to a substitutional B that occupies O sites, can be observed for B-doped samples [36]. This result proves the successful incorporation of B into the TiO_2_ lattice. In Figure 4c,d, four peaks centered at 529.5, 531.5, 532.1, and 532.9 eV, corresponding to lattice oxygen (L_O_), Ti-O in Ti_2_O_3_, a surface adsorbed -OH group, and adsorbed H_2_O (A_O_) [44,45] can be observed in O_1s_ spectra, respectively. In particular, the presence of peaks centered at 531.5 eV is believed to be caused by partially reduced Ti atoms in TiO_2_, due to the connection with neighboring oxygen vacancies (Vo), which suggests the existence of Vo [46,47]. Moreover, the increase in its peak intensity indicates that the Vo content in the samples increases with the increasing content of the B-dopant [29]. Moreover, the content of Vo can be obtained by using the CasaXPS analysis software based on the area-calculation of the fitted peaks, and the result is shown in Figure 4c,d. As can be seen, the ratio of the area under the V_O_ XPS peak (visible at around 531.5 eV) in 10% B-TiO_2_ is the highest, signifying the presence of the highest amount of oxygen vacancies. It can be deduced that the boron in the crystal lattices may reach the highest amount of incorporation, and this sample may show distinct photodegradation activity due to the adjusted band structure. Furthermore, the Ti_2p_ spectra are fitted into four peaks, i.e., 458.3 eV (Ti^4+^ 2p 3/2), 457.3 eV (Ti^3+^ 2p 3/2), 464.0 eV (Ti^4+^ 2p 1/2) and 462.3 eV (Ti^3+^ 2p 1/2), respectively [48]. Moreover, it can be seen that Ti_2p_ peaks (Figure 4e,f) slightly move towards the higher binding energy region from pure-TiO_2_ to 10% B-TiO_2_, then shift towards the lower binding energy region from 10% B-TiO_2_ to 14% B-TiO_2_, indicating the variation of Vo contents with a high electron-attracting effect [41,49]. The presence of Ti^3+^ peaks and the shift further confirm the presence of Vo in all samples and the highest amount of Vo in 10% B-TiO_2_.

### 3.2. Photodegradation Activities of the Catalysts

**Figure 5:**
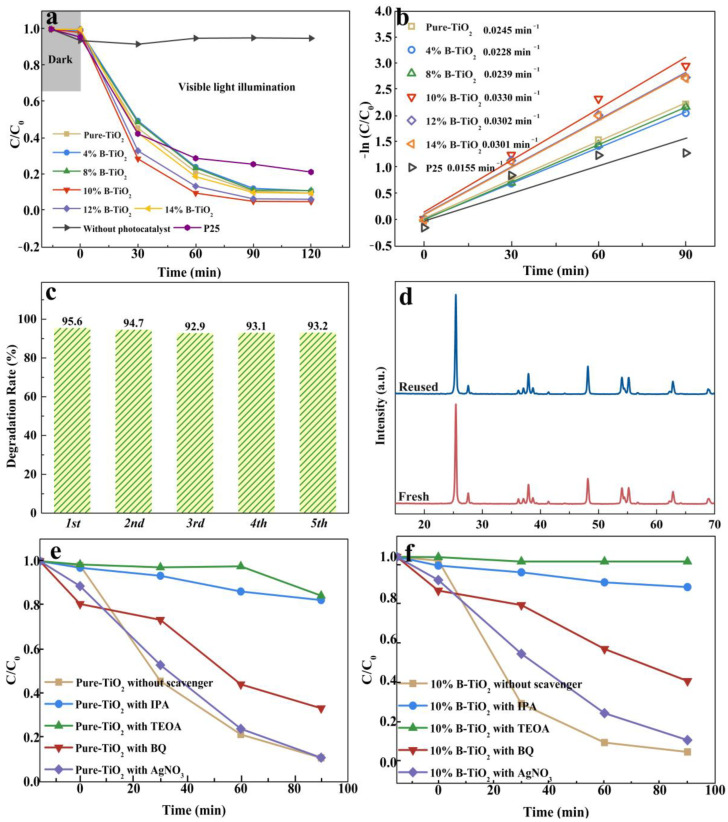
(**a**) RhB photodegradation curves in the absence or presence of prepared catalysts, and (**b**) the kinetics of RhB degradation, (**c**) cycling experiments of 10% B-TiO_2_, (**d**) XRD of the as-prepared and cycled 10% B-TiO_2_, active species trapping experiments over pure-TiO_2_ (**e**) and 10% B-TiO_2_ (**f**) under simulated sunlight illumination.

The RhB photodegradation of the catalysts under simulated sunlight illumination is shown in Figure 5, with commercial P25 and RhB solutions lacking the presence of catalysts as references. No obvious removal of RhB can be observed for the solution without the presence of catalysts. Ten percent B-TiO_2_ shows the highest RhB degradation rate of 94.8% after light illumination for 90 min. The increase in boron doping from 4% B-TiO_2_ to 10% B-TiO_2_ prompted photodegradation activity. However, for the 12% B-TiO_2_ and 14% B-TiO_2_ samples, a significantly decreased degradation efficiency can be observed. From the analysis of degradation kinetics in Figure 5b, the degradation rate with 10% B-TiO_2_ was 0.033 min^−1^, 2.13 times higher than commercial P25. As is known, the chemical stability of photocatalysts plays a crucial role in the application [50]. The cycling test result of the 10% B-TiO_2_ sample is shown in Figure 5c. No obvious deduction (less than 2%) can be observed after five cycles, suggesting that the photocatalyst is relatively stable. From the XRD analysis in Figure 5d, by comparing the position and shape of diffraction peaks, no detectable differences can be seen between the as-prepared and cycled 10% B-TiO_2_, indicating a well-preserved crystalline structure of the catalyst after multiple photocatalytic cycles. Moreover, a few studies on RhB photodegradation performance of TiO_2_-based photocatalysts are summarized in Table 1. The table shows that the degradation efficiency of RhB over the catalyst prepared in this work was markedly improved, indicating that the prepared B-doped TiO_2_ is a promising photocatalyst for RhB degradation.

**Figure 6:**
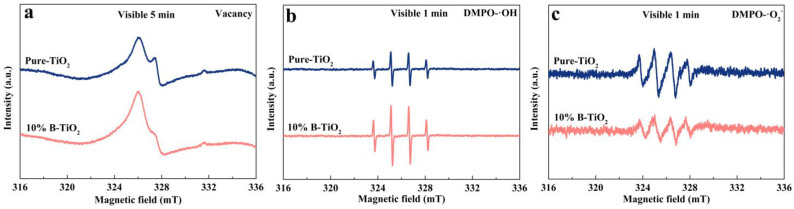
EPR spectra over pure-TiO_2_ and 10% B-TiO_2_ for detecting the Vacancy (**a**), •OH (**b**), and •O_2_^−^ (**c**) radical species under simulated sunlight irradiation.

Figure 5e,f shows the results of the active species trapping experiment of pure-TiO_2_ and 10% B-TiO_2_. Isopropanol (IPA), triethanolamine (TEOA), 1,4-benzoquinone (BQ), and AgNO_3_ were applied to trap hydroxyl radicals (•OH), holes (h^+^), superoxide radicals (•O_2_^−^), and e^−^, respectively. Relative impact of the active species was calculated from rate constants and is presented in Figure S2. As can be seen, for both samples, except for e^−^, the other three active species are found to be critical during the reactions. To further clarify the free active radicals present under simulated sunlight illumination, the characterization of EPR was analyzed and shown in Figure 6. For both samples, •O_2_^−^ and •OH radical are detected after light irradiation. As shown in Figure 6a, characteristic EPR signals at g values of 1.998 (pure-TiO_2_) and 1.997 (10% B-TiO_2_), respectively, are detected. Additionally, the higher signal intensity for 10% B-TiO_2_ can be obviously observed. According to the literature [60], this signal results from unpaired electrons appearing at oxygen vacancies. Therefore, the above results confirm the presence of Vo in both samples and the higher Vo content for 10% B-TiO_2_, which is consistent with the XPS analysis. In Figure 6b, four EPR signals with peak intensities of 1:2:2:1, which can be assigned to DMPO-•OH adducts, were observed for both samples [61]. Additionally, for 10% B-TiO_2_, the signal intensity was apparently higher than that of pure-TiO_2_, indicating more abundant •OH radicals during light reactions. Based on the band structure configuration of the samples, •OH radicals may not be produced from a direct reaction between photogenerated charge carriers and adsorbed H_2_O. Photogenerated electrons can be captured by adsorbed O_2_ and H^+^ in the solution to form H_2_O_2_, which would further decompose into the •OH radicals [62,63]. Therefore, these results further confirm the decrease in the energy-band gap and the obvious positive shift of the VB position of 10% B-TiO_2_. Similarly, quartet signals with peak intensities of 1:1:1:1 attributed to DMPO-•O_2_^−^ adducts were detected for both samples [64] (Figure 6c). The slightly weaker signal intensity further confirms the positive shift of band positions for 10% B-TiO_2_.

### 3.3. Photodegradation Mechanism of the Catalysts

**Figure 7:**
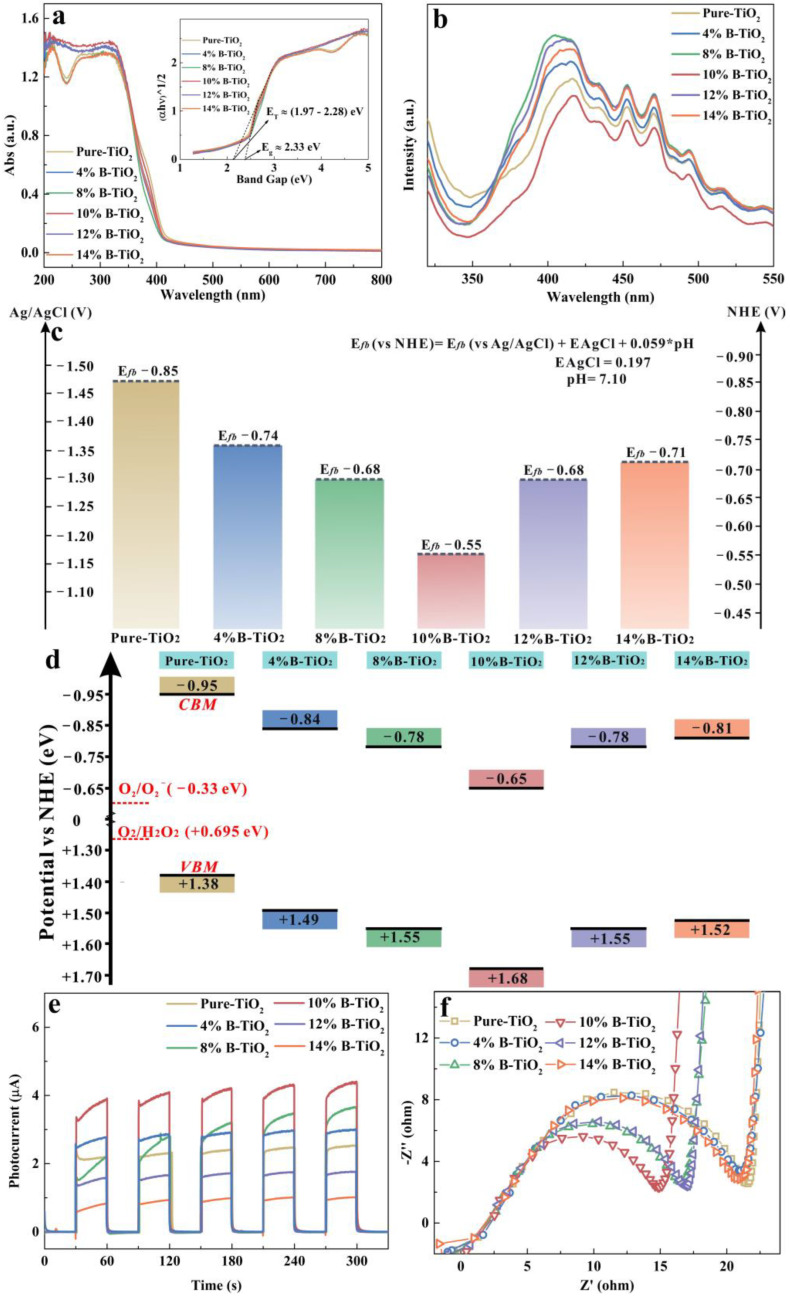
(**a**) UV−Vis diffuse reflectance spectra and Plot of (αhv)^1/2^ versus hν (inset), (**b**) PL spectra, (**c**) comparison of E_fb_ potential variations, (**d**) illustration of band structure variations, (**e**) TP curves and (**f**) EIS results of all prepared catalysts.

The UV–Vis DRS spectra of the catalysts are shown in Figure 7. The light absorption edges show no obvious shift in Figure 7a, with different amounts of B-doping. Furthermore, based on the following Equation (1), the band gaps of the samples were obtained:

αhν = A (hν − Eg)^n⁄2^ (1)

where n = 1, because TiO_2_ belongs to the direct transition semiconductor [65]. The results are shown in the inset image in Figure 7a. There are two band-gap values from all as-prepared samples based on the absorption edge. The inherent band gap (Eg) is 2.33 eV, and the impurity band gap (E_T_) is from 1.97 to 2.28 eV [66]. Furthermore, the CB potentials of the samples were calculated, according to the fact that the CB edge of the n-type semiconductor is 0.10 eV lower than the value of the flat band potential (E_fb_) [66,67]. The E_fb_ value, as shown in Figure 7c, can be obtained through the following Equation (2) [68] based on the Mott-Schottky analysis (Figure S3).

E_fb_ (vs. NHE) = E_fb_ (vs. Ag/AgCl) + E_AgCl_ + 0.059 × pH (2)

Thus, according to E_CB_ = E_VB_ − Eg, the energy band structures of the catalysts were obtained (Figure 7d). As can be seen, the VBM of the sample can be adjusted from 1.38 to 1.68 eV, and the CMB value can be adjusted from −0.95 to 0.65 eV, confirming that the electronic band structure of the prepared catalyst can be effectively dominated by adjusting the content of the B dopant.

From the PL spectra (Figure 7b), the samples show similar emission peaks at around 400 nm. The 10% B-TiO_2_ sample shows the lowest intensity, indicating an enhanced charge separation for 10% B-TiO_2_ with optimized boron doping [69]. Furthermore, the TP response and EIS results are shown in Figure 7e,f. The same trend (firstly increasing and then decreasing) of the TP response and the radicals in EIS can be observed with increasing B-dopant. Especially, the 10% B-TiO_2_ sample shows the highest photocurrent response and the minimum radicals in EIS, which can result from the highest content of oxygen vacancy defects [70]. The above results are consistent with the XPS and photocatalysis degradation studies. In general, it can be concluded that B-doping can adjust the content of oxygen vacancies, and then mediate the band structure to enhance the photocatalytic performance.

Considering the results of XPS and photocatalysis degradation, the reason for the samples with similar band gaps but obviously different photocatalysis performances can be explained as follows. Firstly, all the prepared catalysts including pure-TiO_2_ are identified to be A-TiO_2_/R-TiO_2_ nano-structured heterojunction with the introduction of oxygen vacancy defects. The oxygen vacancies result from the B-dopant or the oxygen-deficit environment during heat-treatment, accompanied by the introduction of defect energy level into the band structure, thus leading to a decrease of the photo-excitation energy and a red-shift of the absorption spectrum edge. Furthermore, defect-energy level served as a springboard to enhance the charge density and the charge separation efficiency. Moreover, the surface-oxygen vacancies worked as reactive sites to obtain quick surface reactions during the photocatalytic process. Secondly, the electronic band structure of the catalyst was mediated, and the optimized band structure was markedly positively shifted, thus more abundant radicals can be produced and participate in the following reactions. Furthermore, according to the literature [71], for B-doped R-TiO_2_, the CB has a decline of approximately 0.24 eV, and the value is approximately 0.48 eV for B doped A-TiO_2_. Moreover, for both B-doped phases, the VB has little shift compared with the pure phases. Combined with the energy-band diagram of pure A-TiO_2_ (CB = +0.012 eV, VB = +2.712 eV) and pure R-TiO_2_ (CB = −0.380 eV, VB = +2.070 eV) [72,73], if the charge transfer pathway follows the type II mode, the reduced reduction potential, which was lower than that of •O_2_^−^/O_2_, would be incapable of causing •O_2_^−^ production, thus the direct Z-scheme A-TiO_2_/R-TiO_2_ heterojunction mechanism is confirmed.

**Figure 8:**
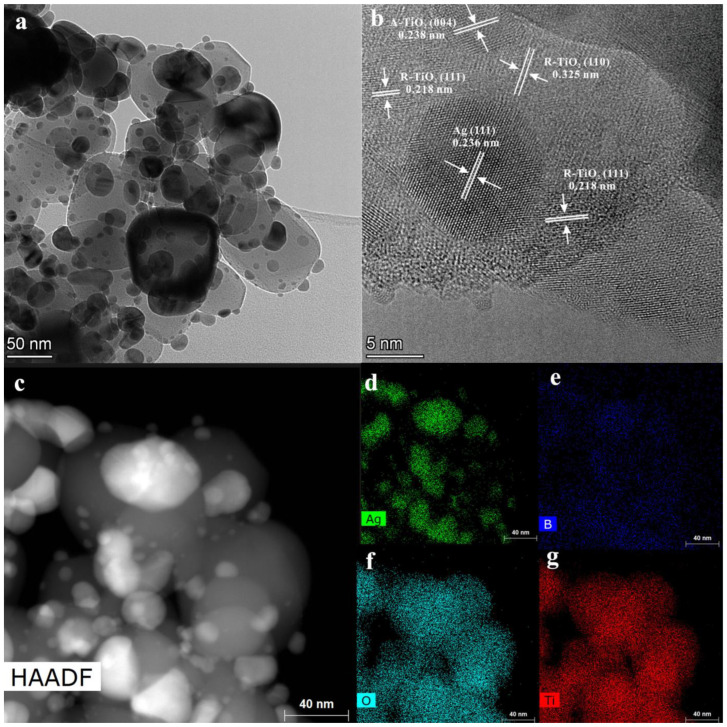
The charge transfer tracking by photo-deposition: (**a**) Low-resolution TEM images, (**b**) high-resolution lattice images, (**c**) HAADF and EDS elemental mapping images of (**d**) Ag, (**e**) B, (**f**) O, (**g**) Ti for the sample 10% B-TiO_2_.

Photodeposition of Ag nanoparticles is known as a convenient method to track the electron-transfer direction. Generally, the Ag nanoparticles are selectively reduced on the site where the photogenerated electron flows to, thus can be used to determine whether the formed heterojunction is Z-scheme or not [74]. Therefore, in order to disclose whether the B doped R–TiO_2_/A–TiO_2_ was a Z-type heterojunction, the charge-transfer tracking experiments, i.e., the e^−^ trapping experiment using AgNO_3_ as the scavenger, were carried out by loading Ag onto the TiO_2_ using the photodeposition method. Figure 8 presents the TEM, HRTEM, and EDS mapping results of the charge-transfer tracking experiment. From the EDS results, uniformly distributed Ag, B, O, and Ti elements can be observed. Moreover, based on the TEM and HRTEM results, the Ag nanoparticles were uniformly distributed, while isolated on R-TiO_2_ and apart from A-TiO_2_. These results demonstrate that the photogenerated electrons were left on the R-TiO_2_, and the photogenerated electrons from A-TiO_2_ could be recombined with the holes from R-TiO_2_, thus forming the Z-type heterojunction.

**Figure 9:**
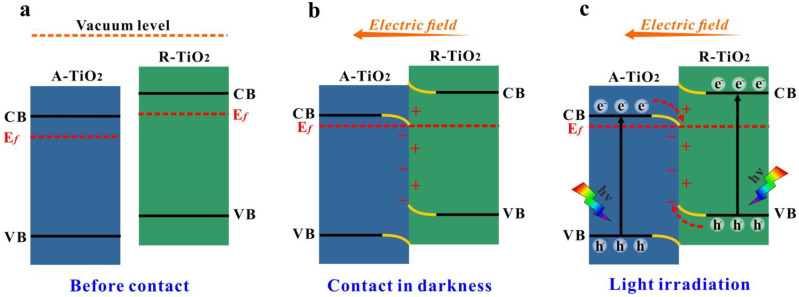
Schematic illustration of formation and charge-transfer processes in the Z-scheme heterojunction of B-doped A-TiO_2_/R-TiO_2_: (**a**) before contact, (**b**) after contact, (**c**) photogenerated charge carrier transfer process in Z-scheme mode.

According to the literatures [71,75], for A-TiO_2_ and R-TiO_2_ with a partially-reduced surface that leads to the remaining oxygen vacancies, the work function of R-TiO_2_ (approximately 4.3 eV) is lower than that of A-TiO_2_ (approximately 4.7 eV). Therefore, the work function of B-doped R-TiO_2_ (R-phase) is deduced to be smaller than that of B-doped A-TiO_2_ (A-phase), both of which are doped to form oxygen vacancies. Therefore, considering the higher CB and VB positions of R-phase and its smaller work function, when the R-phase and the A-phase were in intimate contact, the electrons transferred from the R-phase to the A-phase to get the Fermi levels equilibrated and the internal electric field was formed with the direction from the R-phase to the A-phase. At the same time, the downward and upward band bending was generated at the R-phase/A-phase interface (Figure 9b). Under illumination, electron-hole pairs are produced in both phases. The above formed internal electric field would promote the electrons in the CB of A-phase to recombine with the holes in the VB of R-phase (Figure 9c). Meanwhile, the other charge transfer pathways would be effectively suppressed by the band bending and the built-in electric field. Consequently, the photogenerated electrons in the CB of the R-phase and the holes in the VB of the A-phase are spatially separated and participate in the following redox reactions to produce radicals and degrade organic pollutants.

## 4. Conclusions

In this work, we prepared a direct Z-scheme heterojunction photocatalyst of B-doped A-TiO_2_/R-TiO_2_ with adjustable band structure via B-doping, for which the photocatalytic performances were enhanced by the defect energy level caused by oxygen vacancies, positively shifted-band potential, and synergistically, the Z-type charge transfer pathway. The band structure and surface oxygen-vacancies content can be tailored by controlling the amount of B-dopant. Furthermore, the Z-scheme transfer path between A-TiO_2_ and R-TiO_2_ has promoted the charge carrier separation and improved the redox ability of the sample by retaining the higher redox potential. Moreover, 10% of the B-doping achieved the highest photodegradation performance in this study. The method is applicable to other non-metal ions for enhancing photocatalytic performance.

## Figures and Tables

**Table 1:** Summary of relevant recent works, RhB photodegradation performance of TiO_2_ based heterojunction photocatalysts.

Photocatalyst	C_0_ (mg/L)	Dosage (mg)	Light Source	Degradation	Time (min)	Kinetic Rate (min^−1^)	Ref.
Ag_3_PO_4_/N-TiO_2_	10	20	150 W Xe lamp	94.0%	120	0.0194	[51]
TiO_2_-ZrO_2_	30	200	UV-light	96.5%	150	0.0218	[52]
TiO_2_/ZnO	4.79	40	UV-light	95.0%	120	0.0107	[53]
Ag/ZnO/AgO/TiO_2_	10	30	350 W Xe lamp	99.3%	100	0.0230	[54]
Fe-N-codoped TiO_2_	8	200	500 W Xe lamp	93.13%	250	0.0102	[55]
graphene–TiO_2_	30	375	UV-light	96%	60	0.0280	[56]
B-doped TiO_2_	5	-	500 W Xe lamp	>95%	90	-	[27]
B-doped TiO_2_	4.79	5	Xe lamp	>95%	120	0.038	[36]
A/R-TiO_2_	10	25	UV-light	about 100%	50	-	[57]
Au/A/R-TiO_2_	-	-	UV-light	97%	60	0.0470	[58]
Ce-doped TiO_2_	15	15	UV-Vis light.	99.89%	480	-	[59]
A/R-TiO_2_	10	30	300 W Xe lamp	94.8%	90	0.0370	Thiswork

## Data Availability

The data presented in this study are available on request from the corresponding author.

## Supplementary Materials

The following supporting information can be downloaded at: https://www.mdpi.com/article/10.3390/nano13050794/s1, Figure S1: Particle size distributions of all the prepared catalysts (a) pure TiO_2_, (b) 4% B-TiO_2_, (c) 8% B-TiO_2_, (d) 10% B-TiO_2_, (e) 12% B-TiO_2_, (f) 14% B-TiO_2_; Figure S2: Degradation kinetics of pure TiO_2_ (a) and 10% B-TiO_2_ (b) for active species trapping experiments; Figure S3: Mott-Schottky curves of all the prepared catalysts (a) pure TiO_2_, (b) 4% B-TiO_2_, (c) 8% B-TiO_2_, (d) 10% B-TiO_2_, (e) 12% B-TiO_2_, (f) 14% B-TiO_2_.

## References

[1] Carey J.H., Lawrence J., Tosine H.M. (1976). Photodechlorination of PCB’s in the Presence of Titanium Dioxide in Aqueous Suspensions. Bull. Environ. Contam. Toxicol..

[2] Chen X., Chen Q., Jiang W., Wei Z., Zhu Y. (2017). Separation-Free TiO_2_-Graphene Hydrogel with 3D Network Structure for Efficient Photoelectrocatalytic Mineralization. Appl. Catal. B Environ..

[3] Deng Y., Xing M., Zhang J. (2017). An Advanced TiO_2_/Fe_2_TiO_5_/Fe_2_O_3_ Triple-Heterojunction with Enhanced and Stable Visible-Light-Driven Fenton Reaction for the Removal of Organic Pollutants. Appl. Catal. B Environ..

[4] Ding Y., Yang I.S., Li Z., Xia X., Lee W.I., Dai S., Bahnemann D.W., Pan J.H. (2020). Nanoporous TiO_2_ Spheres with Tailored Textural Properties: Controllable Synthesis, Formation Mechanism, and Photochemical Applications. Prog. Mater. Sci..

[5] Friedmann D., Hakki A., Kim H., Choi W., Bahnemann D. (2016). ChemInform Abstract: Heterogeneous Photocatalytic Organic Synthesis: State-of-the-Art and Future Perspectives. Chem. Inform..

[6] Fu X., Clark L.A., Yang Q., Anderson M.A. (1996). Enhanced Photocatalytic Performance of Titania-Based Binary Metal Oxides: TiO_2_/SiO_2_ and TiO_2_/ZrO_2_. Environ. Sci. Technol..

[7] Espíndola J.C., Cristóvão R.O., Mendes A., Boaventura R.A.R., Vilar V.J.P. (2019). Photocatalytic Membrane Reactor Performance towards Oxytetracycline Removal from Synthetic and Real Matrices: Suspended vs Immobilized TiO_2_-P25. Chem. Eng. J..

[8] Eshete M., Li X., Yang L., Wang X., Zhang J., Xie L., Deng L., Zhang G., Jiang J. (2023). Charge Steering in Heterojunction Photocatalysis: General Principles, Design, Construction, and Challenges. Small Sci..

[9] Hunge Y.M., Yadav A.A., Dhodamani A.G., Suzuki N., Terashima C., Fujishima A., Mathe V.L. (2020). Enhanced Photocatalytic Performance of Ultrasound Treated GO/TiO_2_ Composite for Photocatalytic Degradation of Salicylic Acid under Sunlight Illumination. Ultrason. Sonochem..

[10] Liu C., Li X., Wu Y., Sun L., Zhang L., Chang X., Zhang X., Wang X. (2019). Enhanced Photocatalytic Activity by Tailoring the Interface in TiO_2_–ZrTiO_4_ Heterostructure in TiO_2_–ZrTiO_4_–SiO_2_ Ternary System. Ceram. Int..

[11] Qiang W., Wei L., Shaodan W., Yu B. (2015). Superior Environment Resistance of Quartz Crystal Microbalance with Anatase TiO_2_/ZnO Nanorod Composite Films. Appl. Surf. Sci..

[12] He X., Wu M., Ao Z., Lai B., Zhou Y., An T., Wang S. (2021). Metal–Organic Frameworks Derived C/TiO_2_ for Visible Light Photocatalysis: Simple Synthesis and Contribution of Carbon Species. J. Hazard. Mater..

[13] Xu Q., Zhang L., Yu J., Wageh S., Al-Ghamdi A.A., Jaroniec M. (2018). Direct Z-Scheme Photocatalysts: Principles, Synthesis, and Applications. Mater. Today.

[14] Qi K., Cheng B., Yu J., Ho W. (2017). A Review on TiO_2_-Based Z-Scheme Photocatalysts. Chin. J. Catal..

[15] Jiang L., Yuan X., Zeng G., Liang J., Wu Z., Wang H. (2018). Construction of an All-Solid-State Z-Scheme Photocatalyst Based on Graphite Carbon Nitride and Its Enhancement to Catalytic Activity. Environ. Sci. Nano.

[16] Xu Q., Zhang L., Cheng B., Fan J., Yu J. (2020). S-Scheme Heterojunction Photocatalyst. Chem.

[17] Zeng Z., Jing D., Guo L. (2021). Efficient Hydrogen Production in a Spotlight Reactor with Plate Photocatalyst of TiO_2_/NiO Heterojunction Supported on Nickel Foam. Energy.

[18] Drmosh Q.A., Hezam A., Hendi A.H.Y., Qamar M., Yamani Z.H., Byrappa K. (2020). Ternary Bi_2_S3/MoS_2_/TiO_2_ with Double Z-Scheme Configuration as High Performance Photocatalyst. Appl. Surf. Sci..

[19] Wang Y., Zhu C., Zuo G., Guo Y., Xiao W., Dai Y., Kong J., Xu X., Zhou Y., Xie A. (2020). 0D/2D Co_3_O_4_/TiO_2_ Z-Scheme Heterojunction for Boosted Photocatalytic Degradation and Mechanism Investigation. Appl. Catal. B Environ..

[20] Liu Y., Zeng X., Easton C.D., Li Q., Xia Y., Yin Y., Hu X., Hu J., Xia D., McCarthy D.T. (2020). An in Situ Assembled WO_3_–TiO_2_ Vertical Heterojunction for Enhanced Z-Scheme Photocatalytic Activity. Nanoscale.

[21] Wei T., Zhu Y.-N., An X., Liu L.-M., Cao X., Liu H., Qu J. (2019). Defect Modulation of Z-Scheme TiO_2_/Cu_2_O Photocatalysts for Durable Water Splitting. ACS Catal..

[22] Tang H., Zhang W., Meng Y., Xia S. (2021). A Direct Z-Scheme Heterojunction with Boosted Transportation of Photogenerated Charge Carriers for Highly Efficient Photodegradation of PFOA: Reaction Kinetics and Mechanism. Appl. Catal. B Environ..

[23] Zhang D., Liu W., Wang R., Zhang Z., Qiu S. (2021). Interface Engineering of Hierarchical Photocatalyst for Enhancing Photoinduced Charge Transfers. Appl. Catal. B Environ..

[24] Li Y., Tang Z., Zhang J., Zhang Z. (2017). Fabrication of Vertical Orthorhombic/Hexagonal Tungsten Oxide Phase Junction with High Photocatalytic Performance. Appl. Catal. B Environ..

[25] Qiu Y., Ouyang F. (2017). Fabrication of TiO_2_ Hierarchical Architecture Assembled by Nanowires with Anatase/TiO_2_(B) Phase-Junctions for Efficient Photocatalytic Hydrogen Production. Appl. Surf. Sci..

[26] Wang Y., Zhang W., Wang Z., Cao Y., Feng J., Wang Z., Ma Y. (2018). Fabrication of TiO_2_ (B)/Anatase Heterophase Junctions in Nanowires via a Surface-Preferred Phase Transformation Process for Enhanced Photocatalytic Activity. Chin. J. Catal..

[27] Niu P., Wu G., Chen P., Zheng H., Cao Q., Jiang H. (2020). Optimization of Boron Doped TiO_2_ as an Efficient Visible Light-Driven Photocatalyst for Organic Dye Degradation With High Reusability. Front. Chem..

[28] Ng B., Putri L.K., Kong X.Y., Teh Y.W., Pasbakhsh P., Chai S. (2020). Z-Scheme Photocatalytic Systems for Solar Water Splitting. Adv. Sci..

[29] Chen F., Ma Z., Ye L., Ma T., Zhang T., Zhang Y., Huang H. (2020). Macroscopic Spontaneous Polarization and Surface Oxygen Vacancies Collaboratively Boosting CO_2_ Photoreduction on BiOIO_3_ Single Crystals. Adv. Mater..

[30] Wu J., Li X., Shi W., Ling P., Sun Y., Jiao X., Gao S., Liang L., Xu J., Yan W. (2018). Efficient Visible-Light-Driven CO_2_ Reduction Mediated by Defect-Engineered BiOBr Atomic Layers. Angew. Chem..

[31] Dong C., Lian C., Hu S., Deng Z., Gong J., Li M., Liu H., Xing M., Zhang J. (2018). Size-Dependent Activity and Selectivity of Carbon Dioxide Photocatalytic Reduction over Platinum Nanoparticles. Nat. Commun..

[32] Huang Y., Yu Y., Yu Y., Zhang B. (2020). Oxygen Vacancy Engineering in Photocatalysis. Sol. RRL.

[33] Sarkar A., Khan G.G. (2019). The Formation and Detection Techniques of Oxygen Vacancies in Titanium Oxide-Based Nanostructures. Nanoscale.

[34] You M., Kim T.G., Sung Y.-M. (2010). Synthesis of Cu-Doped TiO_2_ Nanorods with Various Aspect Ratios and Dopant Concentrations. Cryst. Growth Des..

[35] Bilgin Simsek E. (2017). Solvothermal Synthesized Boron Doped TiO_2_ Catalysts: Photocatalytic Degradation of Endocrine Disrupting Compounds and Pharmaceuticals under Visible Light Irradiation. Appl. Catal. B Environ..

[36] Li L., Yang Y., Liu X., Fan R., Shi Y., Li S., Zhang L., Fan X., Tang P., Xu R. (2013). A Direct Synthesis of B-Doped TiO_2_ and Its Photocatalytic Performance on Degradation of RhB. Appl. Surf. Sci..

[37] Wang W.-K., Chen J.-J., Gao M., Huang Y.-X., Zhang X., Yu H.-Q. (2016). Photocatalytic Degradation of Atrazine by Boron-Doped TiO_2_ with a Tunable Rutile/Anatase Ratio. Appl. Catal. B Environ..

[38] Pan X., Yang M.-Q., Fu X., Zhang N., Xu Y.-J. (2013). Defective TiO_2_ with Oxygen Vacancies: Synthesis, Properties and Photocatalytic Applications. Nanoscale.

[39] Pedrosa M., Pastrana-Martínez L.M., Pereira M.F.R., Faria J.L., Figueiredo J.L., Silva A.M.T. (2018). N/S-Doped Graphene Derivatives and TiO_2_ for Catalytic Ozonation and Photocatalysis of Water Pollutants. Chem. Eng. J..

[40] Liu C., Li X., Xu C., Wu Y., Hu X., Hou X. (2020). Boron-Doped Rutile TiO_2_/Anatase TiO_2_/ZrTiO_4_ Ternary Heterojunction Photocatalyst with Optimized Phase Interface and Band Structure. Ceram. Int..

[41] Arif M., Zhang M., Mao Y., Bu Q., Ali A., Qin Z., Muhmood T., Liu X., Zhou B., Chen S.M. (2021). Oxygen Vacancy Mediated Single Unit Cell Bi_2_WO_6_ by Ti Doping for Ameliorated Photocatalytic Performance. J. Colloid Interface Sci..

[42] Quesada-González M., Boscher N.D., Carmalt C.J., Parkin I.P. (2016). Interstitial Boron-Doped TiO_2_ Thin Films: The Significant Effect of Boron on TiO_2_ Coatings Grown by Atmospheric Pressure Chemical Vapor Deposition. ACS Appl. Mater. Interfaces.

[43] Lai Z., Peng F., Wang H., Yu H., Zhang S., Zhao H. (2013). A New Insight into Regulating High Energy Facets of Rutile TiO_2_. J. Mater. Chem. A.

[44] Abdullah S.A., Sahdan M.Z., Nafarizal N., Saim H., Embong Z., Rohaida C.H.C., Adriyanto F. (2018). Influence of Substrate Annealing on Inducing Ti^3+^ and Oxygen Vacancy in TiO_2_ Thin Films Deposited via RF Magnetron Sputtering. Appl. Surf. Sci..

[45] Zhang Y., Chen J., Hua L., Li S., Zhang X., Sheng W., Cao S. (2017). High Photocatalytic Activity of Hierarchical SiO_2_@C-Doped TiO_2_ Hollow Spheres in UV and Visible Light towards Degradation of Rhodamine B. J. Hazard. Mater..

[46] Ząbek P., Eberl J., Kisch H. (2009). On the Origin of Visible Light Activity in Carbon-Modified Titania. Photochem. Photobiol. Sci..

[47] Maarisetty D., Baral S.S. (2020). Defect Engineering in Photocatalysis: Formation, Chemistry, Optoelectronics, and Interface Studies. J. Mater. Chem. A.

[48] Zhang L., Tse M.S., Tan O.K., Wang Y.X., Han M. (2013). Facile Fabrication and Characterization of Multi-Type Carbon-Doped TiO_2_ for Visible Light-Activated Photocatalytic Mineralization of Gaseous Toluene. J. Mater. Chem. A.

[49] Lin J., Heo Y.-U., Nattestad A., Sun Z., Wang L., Kim J.H., Dou S.X. (2014). 3D Hierarchical Rutile TiO_2_ and Metal-Free Organic Sensitizer Producing Dye-Sensitized Solar Cells 8.6% Conversion Efficiency. Sci. Rep..

[50] Yadav A.A., Kang S.-W., Hunge Y.M. (2021). Photocatalytic Degradation of Rhodamine B Using Graphitic Carbon Nitride Photocatalyst. J. Mater. Sci. Mater. Electron..

[51] Khalid N.R., Mazia U., Tahir M.B., Niaz N.A., Javid M.A. (2020). Photocatalytic Degradation of RhB from an Aqueous Solution Using Ag_3_PO_4_/N-TiO_2_ Heterostructure. J. Mol. Liq..

[52] Ruíz-Santoyo V., Marañon-Ruiz V.F., Romero-Toledo R., González Vargas O.A., Pérez-Larios A. (2021). Photocatalytic Degradation of Rhodamine B and Methylene Orange Using TiO_2_-ZrO_2_ as Nanocomposite. Catalysts.

[53] Wang J., Wang G., Wei X., Liu G., Li J. (2018). ZnO Nanoparticles Implanted in TiO_2_ Macrochannels as an Effective Direct Z-Scheme Heterojunction Photocatalyst for Degradation of RhB. Appl. Surf. Sci..

[54] Bian H., Zhang Z., Xu X., Gao Y., Wang T. (2020). Photocatalytic Activity of Ag/ZnO/AgO/TiO_2_ Composite. Phys. E Low-Dimens. Syst. Nanostruct..

[55] Song J., Wang X., Bu Y., Wang X., Zhang J., Huang J., Ma R., Zhao J. (2017). Photocatalytic Enhancement of Floating Photocatalyst: Layer-by-Layer Hybrid Carbonized Chitosan and Fe-N-Codoped TiO_2_ on Fly Ash Cenospheres. Appl. Surf. Sci..

[56] Ali M.H.H., Al-Afify A.D., Goher M.E. (2018). Preparation and Characterization of Graphene–TiO_2_ Nanocomposite for Enhanced Photodegradation of Rhodamine-B Dye. Egypt. J. Aquat. Res..

[57] Zhang X., Lin Y., He D., Zhang J., Fan Z., Xie T. (2011). Interface Junction at Anatase/Rutile in Mixed-Phase TiO_2_: Formation and Photo-Generated Charge Carriers Properties. Chem. Phys. Lett..

[58] Yu Y., Wen W., Qian X.-Y., Liu J.-B., Wu J.-M. (2017). UV and Visible Light Photocatalytic Activity of Au/TiO_2_ Nanoforests with Anatase/Rutile Phase Junctions and Controlled Au Locations. Sci. Rep..

[59] Kasinathan K., Kennedy J., Elayaperumal M., Henini M., Malik M. (2016). Photodegradation of Organic Pollutants RhB Dye Using UV Simulated Sunlight on Ceria Based TiO_2_ Nanomaterials for Antibacterial Applications. Sci. Rep..

[60] Zhou Y., Zhang Q., Shi X., Song Q., Zhou C., Jiang D. (2022). Photocatalytic Reduction of CO_2_ into CH_4_ over Ru-Doped TiO_2_: Synergy of Ru and Oxygen Vacancies. J. Colloid Interface Sci..

[61] Tobaldi D.M., Dvoranová D., Lajaunie L., Rozman N., Figueiredo B., Seabra M.P., Škapin A.S., Calvino J.J., Brezová V., Labrincha J.A. (2021). Graphene-TiO_2_ Hybrids for Photocatalytic Aided Removal of VOCs and Nitrogen Oxides from Outdoor Environment. Chem. Eng. J..

[62] Fónagy O., Szabó-Bárdos E., Horváth O. (2021). 1,4-Benzoquinone and 1,4-Hydroquinone Based Determination of Electron and Superoxide Radical Formed in Heterogeneous Photocatalytic Systems. J. Photochem. Photobiol. Chem..

[63] Li X., Hu Z., Liu J., Li D., Zhang X., Chen J., Fang J. (2016). Ga Doped ZnO Photonic Crystals with Enhanced Photocatalytic Activity and Its Reaction Mechanism. Appl. Catal. B Environ..

[64] Zou Y., Hu Y., Uhrich A., Shen Z., Peng B., Ji Z., Muhler M., Zhao G., Wang X., Xu X. (2021). Steering Accessible Oxygen Vacancies for Alcohol Oxidation over Defective Nb_2_O_5_ under Visible Light Illumination. Appl. Catal. B Environ..

[65] Li N., Li R., Zhao J., Liang L., Yu Y., Kong L., Chen G., Yan B. (2020). Multi-Interface Mn_3_O_4_@ZnO/TiO_2_ with Controllable Charge Transfer Routes for Highly Selective Denitrification under Ultrasonic-Assisted Visible Light Photocatalysis. Chem. Eng. J..

[66] Feng Y., Zhang Z., Zhao K., Lin S., Li H., Gao X. (2021). Photocatalytic Nitrogen Fixation: Oxygen Vacancy Modified Novel Micro-Nanosheet Structure Bi_2_O_2_CO_3_ with Band Gap Engineering. J. Colloid Interface Sci..

[67] Bhandary N., Singh A.P., Kumar S., Ingole P.P., Thakur G.S., Ganguli A.K., Basu S. (2016). In Situ Solid-State Synthesis of a AgNi/g-C_3_N_4_ Nanocomposite for Enhanced Photoelectrochemical and Photocatalytic Activity. ChemSusChem.

[68] An X., Li K., Tang J. (2014). Cu_2_O/Reduced Graphene Oxide Composites for the Photocatalytic Conversion of CO_2_. ChemSusChem.

[69] Chen L., Zhang W., Feng C., Yang Z., Yang Y. (2012). Replacement/Etching Route to ZnSe Nanotube Arrays and Their Enhanced Photocatalytic Activities. Ind. Eng. Chem. Res..

[70] Ângelo J., Magalhães P., Andrade L., Mendes A. (2016). Characterization of TiO_2_-Based Semiconductors for Photocatalysis by Electrochemical Impedance Spectroscopy. Appl. Surf. Sci..

[71] Lin Y., Jiang Z., Zhu C., Hu X., Zhang X., Zhu H., Fan J., Lin S.H. (2013). C/B Codoping Effect on Band Gap Narrowing and Optical Performance of TiO_2_ Photocatalyst: A Spin-Polarized DFT Study. J. Mater. Chem. A.

[72] Wei S., Wang F., Yan P., Dan M., Cen W., Yu S., Zhou Y. (2019). Interfacial Coupling Promoting Hydrogen Sulfide Splitting on the Staggered Type II g-C_3_N_4_/R-TiO_2_ Heterojunction. J. Catal..

[73] Du L., Jin C., Cheng Y., Xu L., An X., Shang W., Zhang Y., Rao X. (2020). Improvement of Antibacterial Activity of Hydrothermal Treated TC4 Substrate through an In-Situ Grown TiO_2_/g-C_3_N_4_ Z-Scheme Heterojunction Film. J. Alloys Compd..

[74] Jiang W., Qu D., An L., Gao X., Wen Y., Wang X., Sun Z. (2019). Purposely Constructing Direct Z-scheme Photocatalyst by Photo-deposition Technique. J. Mater. Chem. A..

[75] Kashiwaya S., Morasch J., Streibel V., Toupance T., Jaegermann W., Klein A. (2018). The Work Function of TiO_2_. Surfaces.

