# Supplementary material for: Oxygen Vacancy Mediated Band-Gap Engineering via B-Doping for Enhancing Z-Scheme A-TiO2/R-TiO2 Heterojunction Photocatalytic Performance
Source: Nanomaterials (Basel). 2023 Feb 21;13(5):794. doi: 10.3390/nano13050794 (PMC10005070; doi:10.3390/nano13050794)
Supplement: Supplementary file 1 [file nanomaterials-13-00794-s001.zip › nanomaterials-2127455-supplementary.pdf]

Supplementary

Oxygen Vacancy Mediated Band-Gap Engineering via B-Doping for Enhancing Z-Scheme A-TiO_2/R-TiO_2 Heterojunction Photocatalytic Performance

Changqing Liu *, Chenggang Xu, Wanting Wang, Long Chen, Xu Li and Yuanting Wu

School of Material Science and Engineering, Shaanxi Key Laboratory of Green Preparation and Functionalization for Inorganic Materials, Shaanxi University of Science and Technology, Xi'an 710021, China
* Correspondence: liu280097311@163.com

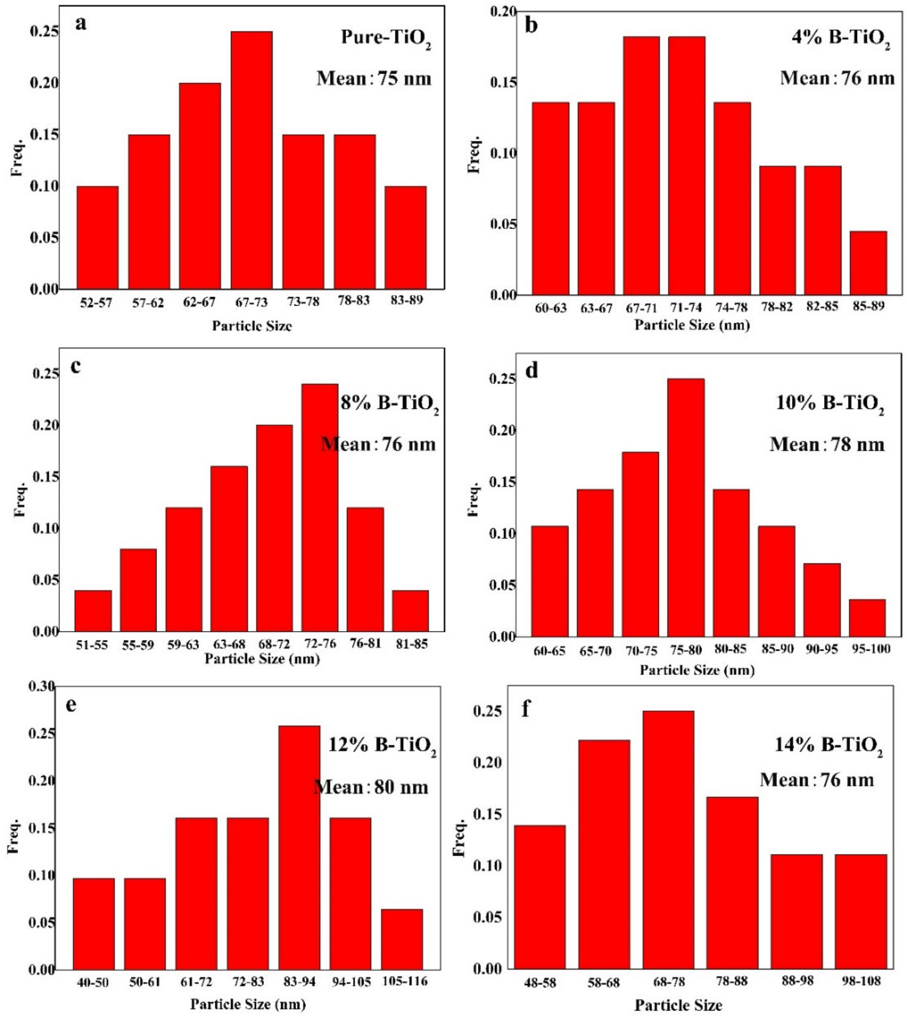

Figure S1. Particle size distributions of all the prepared catalysts (**a**) pure TiO_2, (**b**) 4% B-TiO_2, (**c**) 8% B-TiO_2, (**d**) 10% B-TiO_2, (**e**) 12% B-TiO_2, (**f**) 14% B-TiO_2.

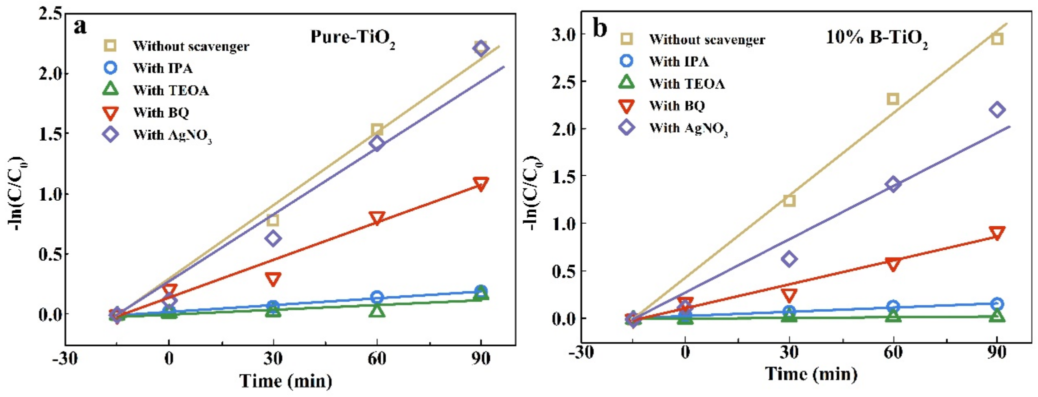

Figure S2. Degradation kinetics of pure TiO_2 (**a**) and 10% B-TiO_2 (**b**) for active species trapping experiments.

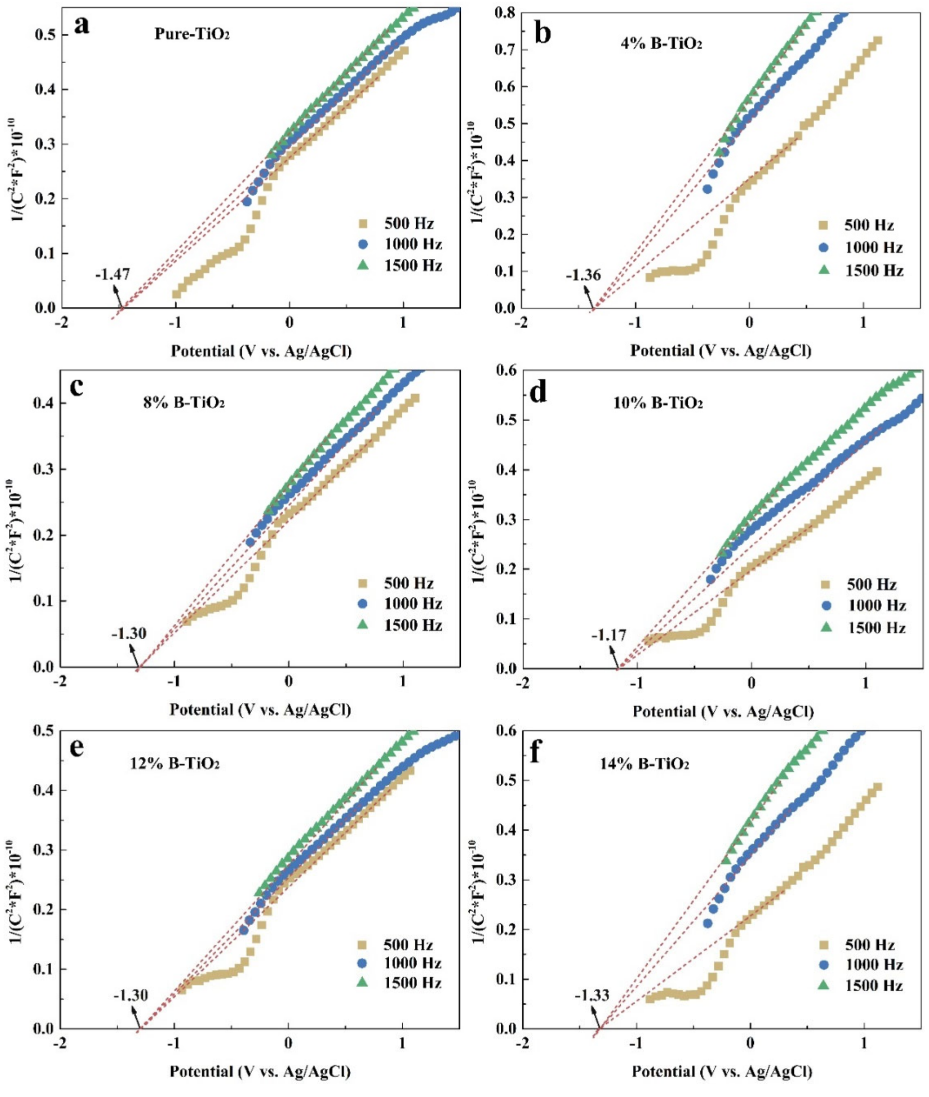

Figure S3. Mott-Schottky curves of all the prepared catalysts (**a**) pure TiO_2, (**b**) 4% B-TiO_2, (**c**) 8% B-TiO_2, (**d**) 10% B-TiO_2, (**e**) 12% B-TiO_2, (f) 14% B-TiO_2.
